# Metabolic Study of Tetra-PEG-Based Hydrogel after Pelvic Implantation in Rats

**DOI:** 10.3390/molecules27185993

**Published:** 2022-09-14

**Authors:** Baoyan Zuo, Mingxue Cao, Xiumei Tao, Xiaoyu Xu, Hongfei Leng, Yali Cui, Kaishun Bi

**Affiliations:** 1 School of Pharmacy, Shenyang Pharmaceutical University, Shenyang 110016, China; 2 Department of Pharmacy, Peking University People’s Hospital, Beijing 100044, China; 3 NKD Pharm Co., Ltd., Beijing 100176, China; 4 Suya Laboratories Co., Ltd., Beijing 102600, China

**Keywords:** tetra-PEG-based hydrogel, radioactive labeling, metabolism, biodistribution

## Abstract

In vivo metabolism of polyethylene glycol (PEG) hydrogels has rarely been studied. In this study, we prepared a chemically crosslinked hydrogel formulation using ^14^C-labeled tetra-armed poly (ethylene glycol) succinimidyl succinate (Tetra-PEG-SS) and ^3^H-labeled crosslinking agent for implantation into the pelvis of Sprague-Dawley (SD) rats. This radioactive labeling technique was used to investigate the radioactivity excretion rates in of feces and urine, the blood exposure time curve, and the radioactivity recovery rate in each tissue over time. We showed that the primary excretion route of the hydrogel was via urine (^3^H: about 86.4%, ^14^C: about 90.0%), with fewer portion through feces (^3^H: about 6.922%, ^14^C: about 8.16%). The hydrogel metabolites exhibited the highest distribution in the kidney, followed by the jejunal contents; The ^3^H and ^14^C radioactivity exposures in the remaining tissues were low. We also showed that the ^3^H and ^14^C radioactivity recovery rates in the blood were usually low (<0.10% g^−1^ at 12 h after implantation), even though, in theory, the hydrogel could be absorbed into the blood through the adjacent tissues. By using a combination of HPLC-MS/MS and offline radioactivity counting method, we established that the tetra-PEG-based hydrogel was mainly metabolized to lower-order PEG polymers and other low-molecular-weight substances in vivo.

## 1. Introduction

Hydrogels are insoluble polymer matrices with a three-dimensional network structure. They are formed by chemical or physical crosslinking of macromonomers [1,2], e.g., covalent bonds, hydrogen bonds, van der Waals force, and so on [3]. With the development of biomedicine and pharmaceutical techniques, the demand for new biomaterials with tailored chemical, physical, and mechanical properties have been continuing to grow [4]. Having the advantages of flexible preparation methods, high water content (typically 70–99%), unique biocompatibility, physical similarity to tissues, and tunable mechanical properties, hydrogels have been widely used in regenerative medicine, cardiology, oncology, immunology, wound healing, pain management, drug delivery and many other fields [1,5].

Bioadhesive hydrogels have multiple functions, such as tissue closure, hemostasis, and anti-adhesion [6,7]. Intraperitoneal tissue adhesion is a common complication of gynecologic surgeries, and the incidence rate in women is about 25–92% [8]. Generally, the key to solving the postoperative adhesion problem is to adopt routine surgical adhesion reduction strategies or apply anti-adhesive agents. PEG-based hydrogels could be an effective and promising anti-adhesive agent for this purpose.

A variety of PEG-based hydrogels have been approved for biomedical applications. For instance, Coseal^®^ (Baxter Healthcare Corporation, Los Angeles, CA, USA), which has been approved by the FDA for its efficacy in sealing vascular wounds, is composed of tetra-armed poly (ethylene glycol) succinimidyl glutarate (tetra-PEG-SG) and tetra-armed poly (ethylene glycol) thiol [9,10,11]. However, since the thiol group can be easily oxidized by the most common oxidants, its “shelf-life” is limited [12,13]. ReSure^®^ Sealant (Ocular Therapeutix, Inc., Bedford, MA, USA), consisting of N-hydroxy succinimide (NHS) modified PEG and trilysine acetate (TLYS), creates a temporary, soft, and lubricious surface barrier to prevent the clearance of corneal incision leakage following the cataract or intraocular lens (IOL) surgery. ReSure^®^ has been found to be superior to sutures [14]. NHS-modified PEG, such as SprayGel (Confluent Surgical Inc., Waltham, MA, USA), is also used as an anti-adhesive in abdominal surgeries [15,16].

**Figure 1:**
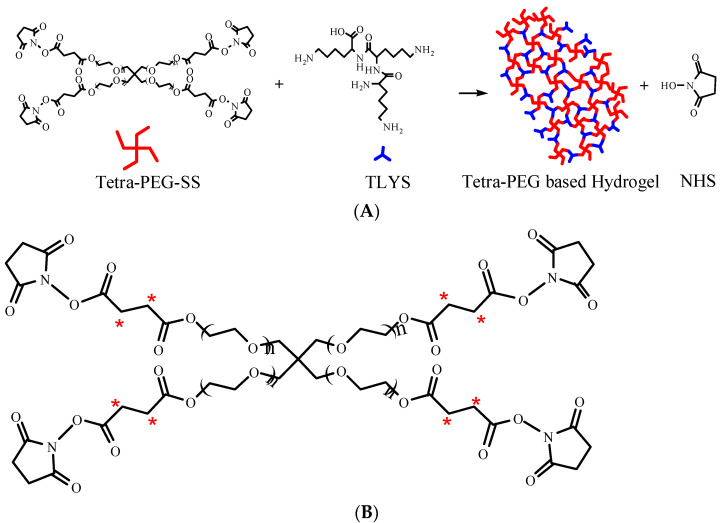
(**A**) Schematic diagram of hydrogel formation. (**B**) Schematic diagram of tetra-PEG-SS radiolabeling (*).

The crosslinking mechanism of tetra-PEG-SS and TLYS forming tetra-PEG-based hydrogels is illustrated in Figure 1A. These PEG-based hydrogels require a specific buffer to dissolve two components containing different reactive groups separately before gel formation. Many studies have reported the involvement of PEG in the metabolic pathways in vivo, where the metabolic and scavenging functions of PEG depend on its molecular weight. PEGs with low molecular weights (<400 Da) are mainly oxidized by ethanol dehydrogenase and acetaldehyde dehydrogenase, producing toxic diacid and hydroxyl acid metabolites that are excreted in urine [17]. In contrast, PEGs with high molecular weights rely on different metabolic pathways, in which the toxicity is very low, and the excretion is through the kidney [18,19]. Although there have been adequate studies on the association between metabolic pathways and PEG monomer, studies on PEG-crosslinked polymers are only a few and limited, especially, concerning the PEG-based hydrogels. Therefore, the impact of the complex three-dimensional structures of PEG-based hydrogels on metabolism warrants further investigation.

In this study, radiolabeling was used to investigate the distribution and excretion of the PEG hydrogels formed by ^14^C-labeled tetra-PEG-SS (Figure 1B, positioning labels of ^14^C-succinic anhydride (2,3-^14^C)), and ^3^H-labeled TLYS (non-positioning) as a crosslinking agent. This hydrogel was implanted into the pelvic cavity of Sprague-Dawley (SD) rats as an anti-adhesion agent. The method of non-positioning labeling for TLYS was to replace ^1^H with ^3^H under certain conditions. This method had the following advantages: fast marker rate, no change of TLYS structure and properties (only atomic swaps), no intermediate impurities or byproducts. The details of positioning labeling process of tetra-PEG-SS were as follows: ^14^C-succinic anhydride was prepared first, and then ^14^C-succinic anhydride was used as a raw material for chemical synthesis according to the synthesis route of Tetra-PEG-SS. Although positioning labels could enable tracking of degradation products containing specific groups or fragments in vivo, positioning labels were difficult to implement for TLYS. Because TLYS had three repeating lysine groups, their structure was simpler, non-positioning labels were easy to implement, and it was possible to track all ^3^H-containing degradation products in vivo.

## 2. Results and Discussion

### 2.1. Properties of Hydrogels

Different tetra-PEG-SS concentrations, molar ratios (tetra-PEG-SS/TLYS), buffer salt concentrations, and buffer salt pH values were selected to prepare hydrogels. The gelation time, swelling ratio, and degradation time of the obtained hydrogels were investigated (Table 1).

We found that the molar ratio of tetra-PEG-SS to TLYS greatly influenced the gelation time, swelling rate, and in vitro degradation time of the hydrogels. When the molar ratio of tetra-PEG-SS to TLYS was 3:4 (to achieve a ratio of active functional groups at 1:1), we observed the shortest gelation time, the lowest swelling rate, and the longest in vitro degradation time. This might be due to the possibility that the active functional groups nearly completely reacted at this ratio, the crosslinking density was high, and/or the curing time was short. Therefore, the formed hydrogel network was dense, and the mechanical strength was high enough to prevent its degradation [20]. The buffer salt concentration had a limited effect on the gelation time, swelling rate, and in vitro degradation time, indicating that the buffer salt at 0.05 mol·L^−1^ could provide sufficient ionic strength for the crosslinking reaction. However, further increasing the salt concentration did not promote crosslinking efficiency. The pH value of the buffer also had a great influence on the gelation time. The crosslinking reaction is essentially a nucleophilic substitution reaction, which can be promoted under alkaline conditions. In general, higher pH values shortened the gelation time. However, there were no significant differences in swelling rate (Fisher’s ex-act test) and degradation time (student’s *t*-test) of hydrogels formed at different pH values, indicating that the pH value only affected the crosslinking reaction rate to a certain extent. The tetra-PEG-SS concentration had a slight influence on the gelation time. Higher tetra-PEG-SS concentrations shortened the gelation time.

Taken together, to achieve a shorter gelation time at the physiological pH (pH7.4, PBS), the molar ratio of tetra-PEG-SS to TLYS should be 3:4 effectively yielding a 1:1 ratio of active functional groups.

### 2.2. Rate of Radioactivity Excretion in Urine and Feces

On the 10th d of implantation of the radiolabeled hydrogel into the pelvic cavity of SD rats, we detected their cumulative radioactive excretion rates in urine and feces. It was found that the total cumulative urine and fecal radioactivity excretion rate of ^3^H was about 93.3%, of which about 86.4% was excreted in urine, and about 6.922% in feces (Table S1 from Supplementary Materials). The total cumulative urine and fecal radioactivity excretion rate of ^14^C was about 98.1%, of which about 90.0% was excreted in urine and about 8.16% in feces. This meant that the ^3^H and ^14^C radioactive elements were mainly excreted in the urine, while a small portion was excreted in feces.

The total cumulative excretion of ^14^C and ^3^H in feces and urine showed the same trend, but the total excretion of ^3^H was lower than that of ^14^C (Figure S1 from Supplementary Materials). That might be because ^3^H was labeled on TLYS. When the hydrogel was degraded in vivo, it released free lysine residues that could be recycled by the organism for protein synthesis or re-entry into other biological processes. Mock surgical group rats were in good condition during this study. There was no cumulative excretion of ^14^C and ^3^H in their feces and urine.

### 2.3. Radioactivity Recovery in Blood

**Figure 2:**
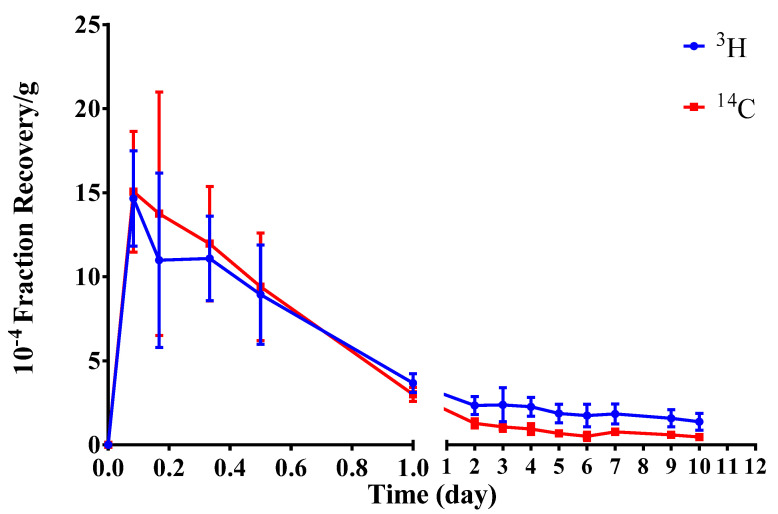
Concentration–time curve of average radioactivity recovery rates in the blood (mean ± SD, *n* = 6). (Student–Newman–Keulsa).

The radioactivity recovery rate in the blood of SD rats was measured within 10d after implantation of radiolabeled hydrogel (Table 2). We constructed the average radioactive recovery rate–time curve for the blood samples (Figure 2).

After the implantation of radiolabeled hydrogel into SD rats, it could be partially absorbed into the blood. The radioactivity recovery rates of both ^3^H and ^14^C in the blood peaked at 2 h after implantation and then decreased over time. Notably, ^3^H and ^14^C radioactivity recovery rates in the blood were very low, reaching < 0.10%∙g^−1^ at 12 h post implantation.

### 2.4. Tissue Distribution

**Figure 3:**
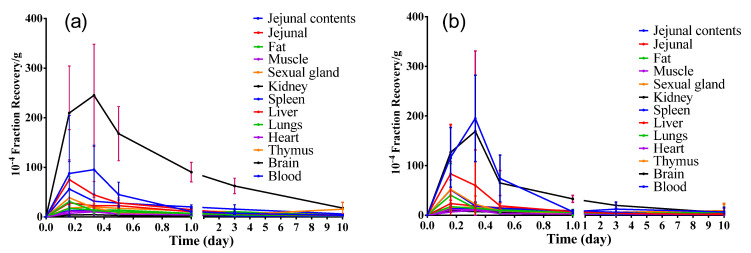
Concentration–time changes of ^3^H (**a**) and ^14^C (**b**) radioactivity in different tissues (mean ± SD, *n*=6). (Student–Newman–Keulsa).

From 0.16 d to 10 d after implantation of the radiolabeled hydrogel into rats, the distribution of ^3^H and ^14^C radioactivity recovery rates in different tissues at different time points were analyzed (Figure 3). Importantly, the highest exposure to the ^3^H and ^14^C radioactivity were in the kidney, followed by the jejunal contents, while exposures in the remaining tissues were low.

The ^3^H and ^14^C radioactivity recovery rates in the jejunal contents and kidney samples showed a small increasing peak at 8 h (0.33 d), then decreasing over time. However, the ^3^H and ^14^C radioactivity recovery rates in other tissues peaked at 4 h (0.16 d), followed by a decrease over time. On the 10th d after hydrogel implantation, the ^3^H and ^14^C radioactivity recovery rates per gram of tissue were both significantly lower than 0.18% of the total implanted radiation dose, indicating that the implantation of the hydrogel was safe after this time point. Based on the above data, we might conclude that the implanted hydrogel in the animal had been completely absorbed, possibly through the contact tissues into the blood, and the kidney might be the main excretory organ for hydrogen metabolites.

The pharmacokinetic (PK) parameters in each tissue were statistically analyzed (Table 3). Following PK parameters were examined in this study: Area under the concentration-time curve (AUC) from time zero extrapolated to 10 d, (AUC_0–10d_), and the maximum concentration (C_max_). It was found that from 0 d to 10 d after implantation of radiolabeled hydrogel in rats, the exposure to ^3^H radioactivity in the kidney tissue was the highest as compared to that of other tissues. The exposure to ^14^C radioactivity in the kidney tissue was also the highest, followed by jejunal contents than that in other tissues. Given the average weight of a normal rat kidney is about 2 g [21,22], the cumulative ^3^H and ^14^C radioactive recovery rates in the kidney were calculated with 12% and 4% of the implanted radiation doses, respectively.

### 2.5. In Vivo Metabolite Analysis

**Figure 4:**
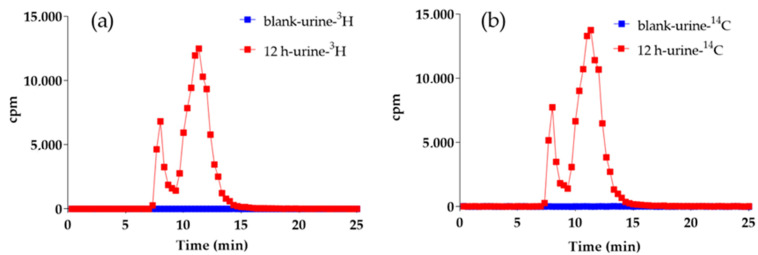
^3^H (**a**) and ^14^C (**b**) radio-chromatograms of blank and 12 h urine.

Rat urine samples were collected continuously for 0 h to 12 h, following the hydrogel implantation, and subsequently analyzed by HPLC-MS/MS methods. Analysis of the offline radioactivity of collected samples revealed no significant radioactivity chromatographic peaks in the control samples. However, in the experimental group, there were two distinct radioactive chromatographic peaks appeared at RT 8.0–10.0 min, and RT 10.0–12.5 min, respectively, at 12 h post implantation. Importantly, the chromatographic profiles of ^3^H and ^14^C radioactivity were identical (Figure 4), suggesting that these peaks were derived from metabolites of the radio-labeled hydrogels after implantation.

**Figure 5:**
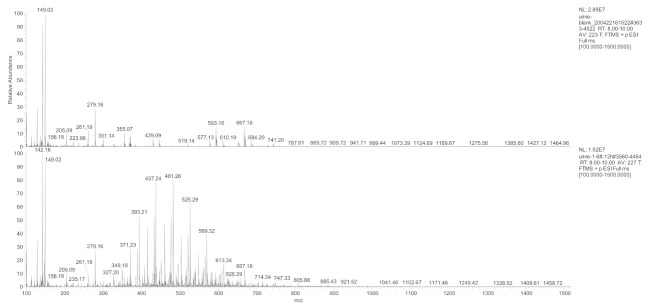
Primary mass spectrum of the control and the experimental group urine samples at the 12 h time point within RT 8.0–10.0 min.

**Figure 6:**
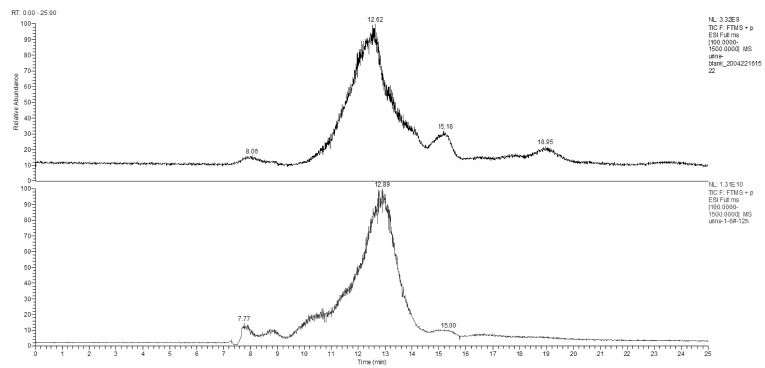
Total ion flow chromatogram of the control and the experimental group urine samples at the 12 h time point.

Different hydrogel-derived metabolites were evaluated by combining the results of the radioactivity chromatographic peaks with the respective mass spectrometry (MS) retention time. The first-stage MS (Figure 5) at *m*/*z* 100–*m*/*z* 1500 within RT 8.0–10.0 min was extracted from the total ion flux (Figure 6) for the control and the experimental urine samples at 12 h. Compared to that 12 h urine, the first-stage mass spectrometry of the control group urine showed a typical polymer mass spectrum in the range of *m*/*z* 400 to *m*/*z* 600 (a series of mass spectra peaks with equal differences in *m*/*z* ).

**Figure 7:**
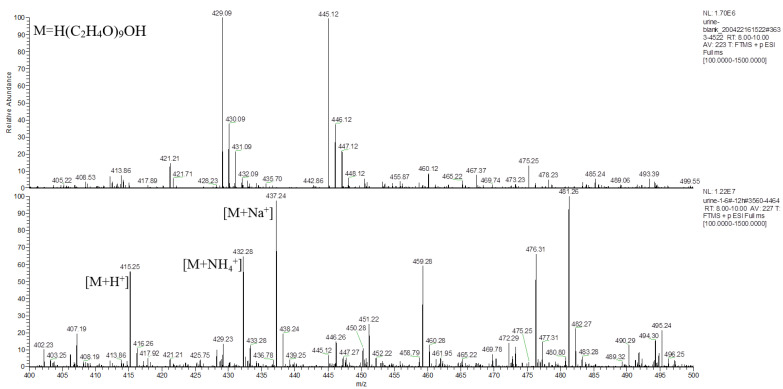
Primary mass spectrum of *m*/*z* 400–500 extracted from the control and the experimental group urine at 12 h time point within RT 8.0–10.0 min.

**Figure 8:**
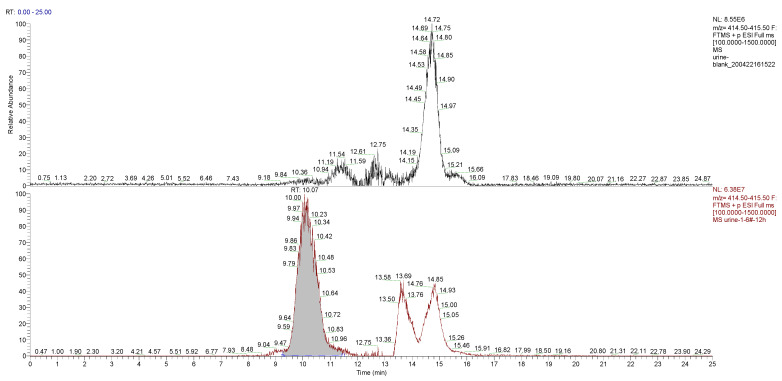
Chromatograms of *m/z* 415.25 were extracted from the control and the experimental group urine samples at the 12 h time point.

The main component of the hydrogel was the tetra-PEG-SS, having a molecular formula of H(C_2_H_4_O)_n_OH, according to the MS analysis. Taking PEG-9 (H(C_2_H_4_O)_9_OH) as an example, analysis of the typical first-stage MS peaks of the control and the experimental group urine samples at 12 h (Figure 7) inferred that *m*/*z* 415.25, *m*/*z* 432.28, and *m*/*z* 437.24 might be the hydrogenation peak, ammonia addition peak, and sodium addition peak of H(C_2_H_4_O)_9_OH, respectively. The chromatograms of the control and the experiment group urine samples at *m*/*z* 415.25 were simultaneously extracted (Figure 8), which showed significantly higher peaks for the experimental samples compared to that of control samples, indicating that *m*/*z* 415.25 was derived from metabolites produced after the labeled hydrogel was implanted, and the products were PEGs with different degrees of polymerizations.

## 3. Materials and Methods

### 3.1. Materials

The ^3^H was used to label TLYS (non-positioning label; radiochemical purity >95%), and the ^14^C was used to label tetra-PEG-SS (positioning label of ^14^C succinic anhydride (2,3-^14^C); radiochemical purity >95%). Both were obtained from the Isotope Institute of China Institute of Atomic Energy (IICIAE; Beijing, China). Tetra-PEG-SS (10K Da), and TLYS were obtained from Beijing Nuokangda Pharma Co., Ltd. (Beijing, China). Pentobarbital sodium was purchased from Skillsmodel Biotechnology (Beijing, China) Co., Ltd. (Beijing, China). Potassium dihydrogen phosphate and sodium hydroxide were purchased from Tianjin Guangfu Technology Development Co., Ltd. (Tianjin, China). Isopropyl alcohol was purchased from Sinopharm Chemical Reagent Co., Ltd. (Shanghai, China). Benzylpenicillin sodium for injection was purchased from Youcare Pharmaceutical Group Co., Ltd. (Beijing, China). Saline was purchased from Shijiazhuang Pharmaceutical Group Co., Ltd. (Shijiazhuang, China).

### 3.2. Preparation and Detection of Hydrogel

Our operating procedure was as follows. First, tetra-PEG-SS, and TLYS were separately dissolved in PBS. Second, these two solutions were mixed immediately and stirred to form the experimental hydrogel. Here, we tested different formulations and conditions, including different tetra-PEG-SS concentrations (0.01 mol∙L^−1^, 0.005 mol∙L^−1^, and 0.002 mol∙L^−1^); different molar ratios (tetra-PEG-SS/TLYS: 2:1, 1:1, 3:4, 1:2, and 1:3); different buffer salt concentrations (0.05 mol∙L^−1^, 0.1 mol∙L^−1^, 0.2 mol∙L^−1^, and 0.3 mol∙L^−1^); and different buffer salt pH values (6.8, 7.4, 7.8 and 9.0).

The ^3^H-labeled TLYS and the ^14^C-labeled tetra-PEG-SS were dissolved in PBS (pH 7.4, 0.01 mol∙L^−1^) separately, and mixed by stirring according to the molar ratio of 3:4 to prepare the hydrogel. Appropriate amounts of radioactive hydrogels were used for the surgical implantation.

#### 3.2.1. Definition of the Gelation Time

The time it took, starting from the mixing of tetra-PEG-SS solution with TLYS solution, until the required hydrogel was formed.

#### 3.2.2. Definition of the Swelling Ratio

According to the gravimetric method [23], 0.3 g of precisely weighed (*W*_1_) hydrogel was dissolved into the 15 mL PBS (pH 7.4, 0.01 mol∙L^−1^), and equilibrated at room temperature for 24 h. Then, the swollen hydrogel was taken out, with the surface moisture removed using a piece of filter paper and accurately weighed (*W*_2_) again. Finally, the swelling ratio (SR; g/g) was calculated according to the following equation:

(1) $$SR=\frac{W_{2}}{W_{1}}$$

where *W*_1_ was the weight of the dried hydrogel, and *W*_2_ was the weight of the swelled hydrogel.

#### 3.2.3. Detection of Degradation Time

The degradation time was detected by the following method: 0.3 g of dried hydrogel was put into the 15mL PBS (pH 7.4, 0.01 mol∙L^−1^), and shaken at 100rpm at 37 °C. Within 24 h, samples were taken out from the oscillator once every hour to observe whether there was any solid gel left. After 24 h, the gelation was observed every 2 h until the solid gel completely disappeared. It was necessary to observe the hydrogel degradation regularly until the hydrogel was completely dissolved; then, the time required for degradation (h) was recorded.

#### 3.2.4. Calibration of the Radioactivity of ^3^H and ^14^C

About 70 mg of radiolabeled hydrogels was placed into a centrifuge tube, then dissolved in 200 μL of 0.1 mol∙L^−1^ NaOH solution by vortexing until it was completely degraded, and then diluted with 1800 μL of purified water. Then, 100 μL of the solution was placed into a combustion boat. After air drying, it was placed into a Harvey OX-501 biological oxidizer. The ^14^CO_2_ and ^3^H_2_O were collected in the scintillation fluid to detect the ^3^H or ^14^C radioactivity, which was defined as the standard of the implant radiation dose. 

According to the calibration testing, the content of ^3^H in each milligram of radiolabeled hydrogel was 1.390 × 106 dpm (0.6263 μCi; 23.17 kBq); and the content of ^14^C in the same radiolabeled hydrogel was 1.250 × 105 dpm (0.05632 μCi; 2.084 kBq). The implanted radiation dose was calculated by subtracting the residual radiation dose in the centrifuge tube from the total implanted radiation dose. 

### 3.3. Animal Grouping and Implantation of the Hydrogel

SD rats (weighing 200–220 g) were supplied by SPF (Beijing, China) Biotechnology Co., Ltd. (Beijing, China). A total 54 8-w-old SD rats (male:female = 1:1) were randomly divided into experimental (*n* = 48) and control (*n* = 6, as the mock surgical group) groups. Their weights were from 200 g to 220 g. All rats were individually housed in a well-ventilated sterile environment. As for the 48 SD rats in the experimental group, they were randomly divided into three sub-groups: 6 rats were in the feces and urine excretion group, 6 rats were in the blood exposure testing group, and 36 rats were in the tissue distribution group. All the experimental groups were composed of 50% male and 50% female rats.

Preparation before operation: rats were intraperitoneally injected with 1% sodium pentobarbital (50 mg·kg^−1^) for anesthesia; then, the head and limbs were fixed.

Surgical procedures: a transverse incision of about 1.0–1.5 cm in length was made in the middle between the umbilicus and the anterior border of the pubis (near the uterine) using a surgical scissor. The skin and muscles were cut. About 70 mg of hydrogel was implanted into the pelvic cavity. After the suture, eighty thousand units of penicillin were applied to each animal to prevent infection. As for the mock surgical group rats, normal salt was implanted into the pelvic cavity site.

### 3.4. Collection and Analysis of Urine and Fecal Samples

At exactly 9:00 a.m. and 9:00 p.m., the urine, and feces of the rats in each group were collected, respectively. Samples were collected every 12 h for the first day, then once a day for the last nine days. During the sample collection, the rat’s tail was lifted with one hand, and urine and feces were collected separately in a sterile test tubes with the other hand. After collection of rat feces weighing 5 g, it was repeatedly mixed in 2 mL of normal saline, and centrifuged at 5000 rpm for 30 min to collect the supernatant for testing.

According to a previous description, the radiation doses of ^3^H and ^14^C in the urine and feces were measured. Briefly, these samples were pipetted into the combustion boat, weighed, dried, and then subjected to oxidative combustion. The ^3^H_2_O or ^14^CO_2_ was pipetted into the ^3^H or ^14^C scintillation fluid, and the radioactivity of ^3^H or ^14^C was counted using the liquid scintillation counting method (LSC), using a liquid scintillation counter (Tri-CarbB2910 TR, Perkin Elmer, Waltham, MA, USA). Finally, based on the sampling size and the total volume of the samples, the radioactivity excretion rate in urine and feces during each time period was calculated using the following equations:

(2) $$Radioactivity\;excretion\;rate\;of\;urine\;\left(\%\right)\frac{\frac{R_{t}}{V_{d}}\times V_{t}\times 100}{R_{i}}$$

(3) $$Radioactivity\;excretion\;rate\;of\;feces\;\left(\%\right)\frac{\frac{R_{t}}{W_{d}}\times W_{t}\times 100}{R_{i}}$$

where *R*_t_ is the sample radioactivity determined, *V*_d_ or *W*_d_ is the volume or weight of the sample detected, *V*_t_ or *W*_t_ is the total amount of samples received at each time point, and *R*_i_ is the implanted radiation dose. 

Additionally, the urine samples were subjected to the HPLC-MS/MS (supplement Equipment parameters) for metabolite analysis in vivo after surgical implantation of the hydrogel. The radioactivity chromatograms were plotted. Possible metabolites were inferred from the MS data, corresponding to the radioactivity chromatogram.

### 3.5. Collection and Analysis of Blood Samples

The sublingual vein blood was collected before and after surgical implantation of the hydrogel at 2 h, 4 h, 8 h, 12 h, and 24 h on the first day, then once a day for the last nine days. These whole blood samples were analyzed according to the above-described method.

### 3.6. Collection and Analysis of Tissue Samples

Rat tissue samples were collected at the following time points: 4 h, 8 h, 12 h, 24 h, 3 d, and 10 d after the surgical implantation of the hydrogel. These animals were deeply anesthetized with isoflurane during the surgery. After anesthesia, rats were sacrificed by jugular exsanguination method. Then, tissue samples were harvested from the brain, jejunal contents, jejunum, muscle, fat, sexual gland (testis or ovary), spleen, kidney, heart, liver, lungs, and thymus. After each tissue specimen was weighed, an equal weight of normal saline was added for tissue homogenization. Finally, specimens were centrifuged at 5000 rpm for 30 min to collect the supernatant for testing using the LSC detection method as described above. The ^3^H and ^14^C radioactivity recovery rates in tissues at each time point were calculated. The area under the radioactivity recovery–time curve (AUC_0–10d_) for each tissue was calculated by the trapezoidal method.

### 3.7. Statistical Analyses 

Statistical analyses were performed using the IBM^®^ SPSS^®^ statistics, version 21.0. The radioactivity positive detection rates were compared using the chi-squared (χ^2^) test (Fisher’s exact test). Differences in the distribution of radioactivity in feces and urine were measured by one-way analysis of variance (ANOVA). The exposure of SD rats’ blood is represented as the mean ± SD of three or more independent experiments. If the data were homogenous, ANOVA, Student–Newman–Keuls (SNK), and Pearson’s correlation analyses were used. While for the non-homogenous data, Kruskal–Wallis and Games–Howell tests, and a Spearman’s correlation analysis, were used. Statistical significance was defined at a *p*-value < 0.05.

This study was conducted in accordance with the Declaration of Helsinki, and the recommendations in the Guide for the Care and Use of Laboratory Animals of the National Institutes of Health. The protocol was approved by the Committee on the Ethics of Animal Experiments of Suya Laboratories Co., Ltd. (Beijing, China). (approval number: SY-2020-03). All surgeries were performed under anesthesia, and great care was taken to minimize suffering.

## 4. Conclusions

In this study, a radiolabeled tracer technology was used to investigate the fecal and urine radioactivity excretion rates, the blood exposure–time curve, and the radioactive recovery rate in each tissue over time by implanting tetra-PEG-based hydrogel in rat pelvis. We found that significant amounts of ^3^H and ^14^C-labeled radioactive metabolites were excreted through the urine, while a small portion was excreted through feces. Our observations were consistent with the reported PEG excretion route [18,19]. ^3^H and ^14^C were almost completely excreted 10 d after the hydrogel implantation. After implantation, the hydrogel could be partially absorbed into the blood through the adjacent tissues, but the ^3^H and ^14^C radioactivity recovery rates in the blood were low. The ^3^H and ^14^C radioactivity exposure in the kidney tissue was the highest, followed by the jejunal contents but the ^3^H and ^14^C radioactive exposure in the remaining tissues was low too.

The metabolites of tetra-PEG-SS crosslinked hydrogels in SD rats were analyzed using the HPLC-MS/MS method combined with offline radioactivity counting. We showed that the metabolites of the hydrogels were mainly lower-order PEG polymers and other substances with low molecular weights.

However, the current method possesses certain limitations. For example, ^3^H labeling on TLYS was a non-positional marker, and TLYS could form a variety of fragments with ^3^H after degradation in vivo. Therefore, the results of ^3^H metabolism and excretion might only reflect a general metabolic level, with poor specificity. Some of these fragments containing ^3^H can re-participate in the biosynthesis of other substances in the body, resulting in a longer metabolism time for ^3^H than for ^14^C.

## Figures and Tables

**Table 1:** Investigation results of hydrogel properties (mean ± SD, *n* = 3).

No.	C_Tetra-PEG-SS_ (mol∙L^−1^)	Molar Ratio (Tetra-PEG-SS/TLYS)	C_Buffer_ (mol∙L^−1^)	pH	Gelation Time (s)	Swelling Ratio	Degradation Time (h)
1	0.01	2:1	0.05	7.4	150 ± 6	4.01 ± 0.15	17 ± 1
2	0.01	1:1	0.05	7.4	34 ± 4	3.26 ± 0.11	40 ± 2
3	0.01	3:4	0.05	7.4	19 ± 2	1.92 ± 0.08	48 ± 2
4	0.01	1:2	0.05	7.4	44 ± 3	2.45 ± 0.12	32 ± 0
5	0.01	1:3	0.05	7.4	80 ± 4	5.96 ± 0.08	17 ± 1
6	0.01	1:1	0.1	7.4	16 ± 2	1.86 ± 0.05	48 ± 2
7	0.01	1:1	0.2	7.4	28 ± 2	1.89 ± 0.04	48 ± 2
8	0.01	1:1	0.3	7.4	24 ± 2	1.93 ± 0.12	48 ± 2
9	0.005	1:1	0.1	7.4	38 ± 3	2.03 ± 0.12	48 ± 4
10	0.002	1:1	0.1	7.4	47 ± 2	1.96 ± 0.04	48 ± 2
11	0.01	1:1	0.1	6.8	248 ± 15	2.05 ± 0.08	48 ± 2
12	0.01	1:1	0.1	7.8	15 ± 2	1.91 ± 0.11	48 ± 2
13	0.01	1:1	0.1	9.0	12 ± 2	1.88 ± 0.05	48 ± 2

**Table 2:** The average radioactivity recovery concentrations of ^3^H and ^14^C in blood samples were collected at each time point (mean ± SD, *n* = 6).

Day	Blood Recovery Concentration (10^−4^∙g^−1^)	
	^3^H	^14^C
0.083	14.66 ± 2.833	15.06 ± 3.586
0.167	10.99 ± 5.184	13.75 ± 7.236
0.333	11.09 ± 2.509	11.96 ± 3.399
0.5	8.94 ± 2.952	9.41 ± 3.199
1	3.673 ± 0.555	3.006 ± 0.420
2	2.338 ± 0.535	1.283 ± 0.270
3	2.384 ± 1.009	1.063 ± 0.260
4	2.262 ± 0.562	0.943 ± 0.316
5	1.866 ± 0.547	0.681 ± 0.165
6	1.746 ± 0.678	0.508 ± 0.244
7	1.842 ± 0.595	0.781 ± 0.151
9	1.583 ± 0.507	0.593 ± 0.177
10	1.376 ± 0.503	0.470 ± 0.160

**Table 3:** Pharmacokinetic parameters in each tissue (mean ± SD, *n* = 6).

Tissues	^14^C			^3^H		
	C_max_ (10^−4^∙g^−1^)	AUC_0–10d_ (10^−4^/g∙d)	T_max_ (d)	C_max_ (10^−4^∙g^−1^)	AUC_0–10d_ (10^−4^/g∙d)	T_max_ (d)
Jejunal Contents	222.9 ± 58.37	164.5 ± 61.40	0.2733 ± 0.0878	141.7 ± 99.7	104.0 ± 29.90	0.3017 ± 0.0694
Jejunal	86.1 ± 98.3	61.98 ± 26.87	0.1883 ± 0.0694	78.08 ± 99.5	88.5 ± 19.27	0.2167 ± 0.1388
Fat	46.23 ± 61.13	54.37 ± 42.20	1.942 ± 3.951	35.15 ± 48.08	45.30 ± 17.49	0.3583 ± 0.1280
Muscle	11.74 ± 8.46	24.28 ± 6.064	0.2450 ± 0.0931	11.15 ± 5.817	43.12 ± 6.663	0.2167 ± 0.0878
Sexual gland	52.09 ± 56.00	63.30 ± 50.88	0.2167 ± 0.1388	39.64 ± 49.25	95.0 ± 54.14	0.2167 ± 0.0878
Kidney	205.8 ± 138.9	219.7 ± 37.77	0.2450 ± 0.0931	284.3 ± 85.7	589.4 ± 70.01	0.2450 ± 0.0931
Spleen	51.56 ± 63.56	60.35 ± 11.05	0.2167 ± 0.1388	57.53 ± 54.43	142.2 ± 47.87	0.2450 ± 0.1422
Liver	24.10 ± 5.620	59.00 ± 15.12	0.2167 ± 0.1388	28.43 ± 3.861	73.94 ± 11.97	0.1883 ± 0.0694
Lungs	20.39 ± 5.202	35.04 ± 10.03	0.2733 ± 0.1388	18.71 ± 2.187	77.58 ± 17.17	0.3017 ± 0.1671
Heart	14.48 ± 3.938	30.94 ± 13.45	0.2167 ± 0.0878	13.77 ± 1.645	64.57 ± 13.98	0.7183 ± 1.121
Thymus	24.86 ± 8.96	79.61 ± 62.25	1.913 ± 3.964	22.97 ± 3.781	88.6 ± 18.45	0.3300 ± 0.1075
Brain	12.17 ± 5.805	42.66 ± 35.95	1.885 ± 3.978	5.611 ± 1.881	34.22 ± 9.35	0.8017 ± 1.125
Blood	17.64 ± 5.353	17.79 ± 4.014	0.2733 ± 0.1388	16.31 ± 3.191	26.96 ± 3.916	0.2733 ± 0.1388

## Data Availability

The data that support the findings of this study are available on request from the corresponding author. The data are not publicly available due to privacy or ethical restrictions.

## Supplementary Materials

The following are available online at https://www.mdpi.com/article/10.3390/molecules27185993/s1, Figure S1: Cumulative ^3^H and ^14^C radioactive excretion curve of urine and feces within 10 days, Table S1: Cumulative radioactivity excretion rate of urine, feces and total, Supplement equipment parameters: Chromatographic conditions.

## Sample Availability

Samples were consumed during analysis and are thus not available.
