# Supplementary material for: Metabolic Study of Tetra-PEG-Based Hydrogel after Pelvic Implantation in Rats
Source: Molecules. 2022 Sep 14;27(18):5993. doi: 10.3390/molecules27185993 (PMC9501824; doi:10.3390/molecules27185993)
Supplement: Supplementary file 1 [file molecules-27-05993-s001.zip › molecules-1800907-supplementary.pdf]

molecules

MDPI

Supplementary Materials

Metabolic Study of Tetra-PEG-Based Hydrogel after Pelvic Implantation in Rats

Baoyan Zuo [1,†], Mingxue Cao [2,†], Xiumei Tao [3], Xiaoyu Xu [3], Hongfei Leng [3], Yali Cui [4] and Kaishun Bi [1,*]

[1] School of Pharmacy, Shenyang Pharmaceutical University, Shenyang 110016, China
[2] Department of Pharmacy, Peking University People's Hospital, Beijing 100044, China
[3] NKD Pharm Co. Ltd., Beijing 100176, China
[4] Suya Laboratories Co. Ltd., Beijing 102600, China
* Correspondence: kaishunbi.syphu@gmail.com
† These authors contributed equally to this work.

Table S1. Cumulative radioactivity excretion rate of urine, feces and total (mean±SD, *n* = 6).

	Day	Urine	Feces	Total
**Cumulative ^{3}H radioactive excretion rate (%)**	0.5	69.90 ± 9.67	0.000 ± 0.000	69.90 ± 9.67
	1	76.84 ± 9.69	0.952 ± 0.703	77.79 ± 9.56
	2	80.3 ± 9.28	3.281 ± 1.265	83.6 ± 9.61
	3	82.3 ± 9.41	4.630 ± 1.945	86.9 ± 9.71
	4	83.1 ± 9.56	5.402 ± 2.130	88.5 ± 9.95
	5	83.5 ± 9.58	5.751 ± 2.213	89.3 ± 9.95
	6	84.6 ± 9.94	6.168 ± 2.375	90.8 ± 10.04
	7	85.0 ± 9.86	6.380 ± 2.406	91.3 ± 9.97
	8	85.5 ± 10.05	6.552 ± 2.425	92.0 ± 10.15
	9	86.1 ± 10.47	6.744 ± 2.409	92.8 ± 10.54
	10	86.4 ± 10.6	6.922 ± 2.396	93.3 ± 10.67
**Cumulative ^{14}C radioactive excretion rate (%)**	0.5	79.16 ± 15.40	0.000 ± 0.000	79.16 ± 15.40
	1	85.0 ± 14.77	1.531 ± 1.294	86.5 ± 14.28
	2	87.3 ± 15.39	4.885 ± 2.573	92.1 ± 15.30
	3	88.2 ± 15.33	6.592 ± 3.331	94.8 ± 15.61
	4	88.6 ± 15.25	7.141 ± 3.588	95.7 ± 15.61
	5	88.7 ± 15.23	7.401 ± 3.650	96.1 ± 15.60
	6	88.9 ± 15.26	7.767 ± 3.977	96.7 ± 15.81
	7	89.1 ± 15.25	7.898 ± 4.017	97.0 ± 15.79
	8	89.5 ± 14.86	7.969 ± 4.001	97.4 ± 15.37
	9	89.7 ± 14.54	8.08 ± 3.997	97.8 ± 14.99
	10	90.0 ± 14.24	8.16 ± 4.002	98.1 ± 14.65

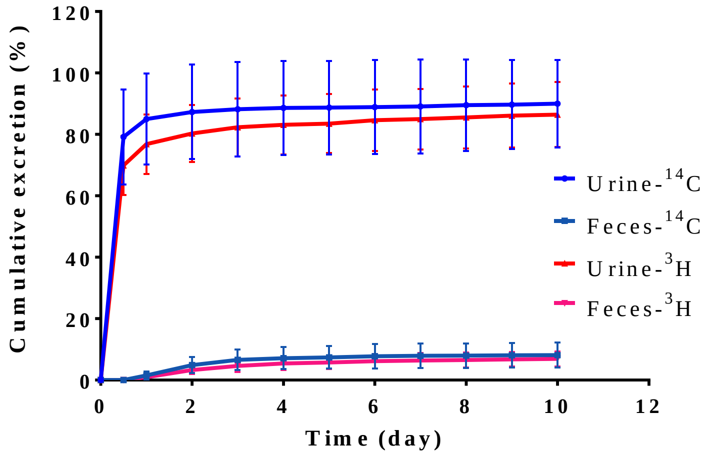

Figure S1. Cumulative ^{3}H and ^{14}C radioactive excretion curve of urine and feces within 10 days (mean ± SD, *n* = 6).

Supplement equipment parameters: Chromatographic conditions

1. The sample analysis was performed using an UltiMate3000 liquid chromatography/ mass spectrometer (UltiMate3000 UPLC-Q Exactive Orbitrap, Thermo, Waltham, MA, USA) with the following parameters:
2. Column: TSK gel 2500 PWXL TOSOH; isocratic elution; flow rate: 0.7mL·min^{-1}; mobile phase A: pure water (0.1% formic acid); mobile phase B: acetonitrile (0.1% formic acid); A: B=1:1; total time: 25 min; and collection duration of each tube: 20s.
3. Mass spectrum conditions: Sheath gas flow rate 40，Aux gas flow rate 10，Spray voltage 3.5kV，Capillary temperature 325℃.
4. Scan type: Full mass, Polarty: Positive, Resolution: 70,000, Scan range: 100-1500 m/z; dd-MS2: Discovery, Resolution: 17,500, (N)CE: 20, 40 and 60.
